# Results of Optimize: A Cluster Randomized Trial of Patient, Family, and Provider Education in Primary Care

**DOI:** 10.1093/geroni/igab046.1560

**Published:** 2021-12-17

**Authors:** Ariel Green, Elizabeth Bayliss, Susan Shetterly, Melanie Drace, Jonathan Norton, Emily Reeve, Orla Sheehan, Cynthia Boyd

**Affiliations:** 1 Johns Hopkins University School of Medicine, Baltimore, Maryland, United States; 2 Kaiser Permanente Colorado, Denver, Colorado, United States; 3 Johns Hopkins University, Baltimore, Maryland, United States; 4 University of South Australia, Adelaide, South Australia, Australia; 5 Johns Hopkins University School of Medicine, Johns Hopkins University, Maryland, United States

## Abstract

Individuals with cognitive impairment frequently have multiple chronic conditions (MCC), increasing their risk for polypharmacy and associated adverse outcomes. Optimizing medications through deprescribing (reducing or stopping the use of inappropriate medications or medications unlikely to be beneficial) may improve outcomes for this population. Optimize was a pragmatic, 12-month cluster-randomized trial of deprescribing in primary care within a not-for-profit integrated delivery system. Participants were age 65+ with dementia or mild cognitive impairment (MCI), 2+ chronic conditions, and 5+ chronic medications. The intervention consisted of a deprescribing educational brochure for patients/caregivers, and Tip Sheets for primary care clinicians. Outcomes were the number of chronic medications and presence of potentially inappropriate medications (PIM). In total, 1,433 patients received, and 1,579 control clinic patients would have been eligible to receive, the intervention (N=3,012). After 6 months, mean estimates of chronic medications were 6.23 in the intervention group and 6.33 in the control group adjusting for baseline counts, age, and gender (p=0.13). Excluding those without complete 90 days follow-up increased the adjusted effect size to 0.14 (p=0.08). In sub-analyses of individuals with 7+ medications at baseline (N= 1,434), the adjusted effect size was 0.19 (p=0.07) at 6 months and 0.21 (p=0.045) when excluding those without complete 90 days’ follow-up. Change in proportions of PIM did not differ between intervention and control groups. An educational intervention for patients, caregivers and clinicians may prompt reductions in chronic medications. The relatively small effect size highlights the complexity of medication management for individuals with dementia or MCI and MCC.

